# Overlapping Regions in HIV-1 Genome Act as Potential Sites for Host–Virus Interaction

**DOI:** 10.3389/fmicb.2016.01735

**Published:** 2016-11-04

**Authors:** Deeya Saha, Soumita Podder, Tapash C. Ghosh

**Affiliations:** ^1^Bioinformatics Centre, Bose InstituteKolkata, India; ^2^Department of Microbiology, Raiganj UniversityRaiganj, India

**Keywords:** structural disorder, short linear motifs, gene overlapping, host–pathogen interaction, HIV-1, protein phosphorylation

## Abstract

More than a decade, overlapping genes in RNA viruses became a subject of research which has explored various effect of gene overlapping on the evolution and function of viral genomes like genome size compaction. Additionally, overlapping regions (OVRs) are also reported to encode elevated degree of protein intrinsic disorder (PID) in unspliced RNA viruses. With the aim to explore the roles of OVRs in HIV-1 pathogenesis, we have carried out an in-depth analysis on the association of gene overlapping with PID in 35 HIV1- M subtypes. Our study reveals an over representation of PID in OVR of HIV-1 genomes. These disordered residues endure several vital, structural features like short linear motifs (SLiMs) and protein phosphorylation (PP) sites which are previously shown to be involved in massive host–virus interaction. Moreover, SLiMs in OVRs are noticed to be more functionally potential as compared to that of non-overlapping region. Although, density of experimentally verified SLiMs, resided in 9 HIV-1 genes, involved in host–virus interaction do not show any bias toward clustering into OVR, *tat* and *rev* two important proteins mediates host–pathogen interaction by their experimentally verified SLiMs, which are mostly localized in OVR. Finally, our analysis suggests that the acquisition of SLiMs in OVR is mutually exclusive of the occurrence of disordered residues, while the enrichment of PPs in OVR is solely dependent on PID and not on overlapping coding frames. Thus, OVRs of HIV-1 genomes could be demarcated as potential molecular recognition sites during host–virus interaction.

## Introduction

Overlapping regions (OVR) where one DNA sequence codes for multiple proteins with different reading frames, are omnipresent in diverse life forms, for instance, it has been observed in the genomes of acellular obligate parasites like virus (Barrell et al., 1976; Pavesi, 2006; Chirico et al., 2010; Simon-Loriere et al., 2013), prokaryotes including archaea (Saha et al., 2016) and eubacteria (Saha et al., 2016) and subsequently in complex eukaryotes such as human (Veeramachaneni et al., 2004; Makalowska et al., 2007; Sanna et al., 2008). Overlapping genes had been widely studied as a mechanism to minimize genome size in prokaryotes (Sakharkar et al., 2005; Sabath et al., 2013; Saha et al., 2015, 2016). Genomesize minimization again was attributed to environmental stimuli such as a rise in temperature (Saha et al., 2015) or in response to life history traits such as faster growth rates (Saha et al., 2016) in prokaryotes. Similar to the prokaryotes, recent studies have widely employed gene overlapping as a phenomenon of generating genetic novelty in RNA viruses (Pavesi, 2006). Moreover, the low fidelity of replication in RNA viruses compelled them to execute compact genomic structure since the longer genome size increases the chances of higher mutational load (Holland et al., 1982; Domingo, 1997). Hence, gene overlapping act as an effective mechanism of compacting genome size (Chirico et al., 2010). Simon-Loriere et al. (2013) showed that OVRs in RNA viruses possessed lower rates of evolution as compared to non-overlapping counterparts. This may in turn constrain the adaptive potential of RNA viruses (Elena et al., 2006; Belshaw et al., 2008). In addition to that OVRs also could encode proteins with novel features. In another study, Rancurel et al. (2009) reported that OVRs in unspliced RNA virus genomes encode significant amount of structural disorder. These intrinsically disordered proteins play an essential part in viral proteome as they are typically enriched with Short linear motifs (SLiMs) (Fuxreiter et al., 2007), post-translational modification sites (PTMs; Gao et al., 2013; Kurotani et al., 2014), and proteolytic cleavage sites (Fan et al., 2014). Viruses extensively use these sites to interact and hijack host cellular machinery and thus intrinsically disordered proteins help in viral spread and invasion in host system (Hagai et al., 2014).

Human Immunodeficiency Virus (HIV) is a retrovirus that has evolved from simian immunodeficiency virus (SIV; Sharp and Hahn, 2011) causing acquired immunodeficiency syndrome (AIDS) in human. Though HIV-1 encodes only eight proteins, yet is a potential human pathogen hijacking the entire human cellular machinery. Significant volume of research concentrates to explore how do retroviruses like HIV-1 with such small genome can evade the host’s complex immune system (Johnson and Desrosiers, 2002; Castiglione and Bernaschi, 2005). Studies on human HIV-1 interactome revealed that HIV-1 generally targets host hub like proteins (Evans et al., 2009; Halehalli and Nagarajaram, 2015). Targeting host hubs could intervene with many other interactions essential for cellular maintenance and survival. Although, it is known that HIV-1 proteins can interact and regulate a number of host proteins, there is a dearth of research explaining the exact structural properties of HIV-1 proteins that help the virus to fascinate multiple interactions with host machinery. It has been previously explored that HIV-1 genome contains considerable numbers of coding regions that are overlapping in nature (Mayrose et al., 2013). However, if there is any structural specialty of proteins encoded by these OVRs which in turn facilitates HIV-1 interaction with host machinery remains to be elucidated. Hence, our study aims to explore whether OVRs in HIV-1 genome encode intrinsically disordered proteins or not, if yes, then how these disordered regions are utilized to successfully invade host cellular machinery.

## Materials and Methods

### Preparation of Overlapping Gene Dataset from Genomes of HIV-1 Group M Subtype

We chose HIV-1 group M as our model system of study because the group M subtype is the most predominant HIV-1 strains which are responsible for AIDS pandemic. HIV-1 group M reference sequences were downloaded from http://www.hiv.lanl.gov. The list of HIV-1 group M subtype genomes used in this study is provided in **Supplementary Table 1**. All of these group M subtype genomes were of non-recombinant types. The genomic coordinates of each gene from each of the HIV-1 genomes were downloaded from GenBank. From these genomic coordinates, we have identified regions of gene to gene overlap in the respective genomes (Sabath et al., 2008). We considered those regions as true overlaps where a two adjacent genes involved in overlapping are in different reading frames. The details of overlapping gene pairs of each HIV-1 genome used in this study, along with their genomic coordinates and reading frames are enlisted in **Supplementary Table 1**.

### Prediction of Protein Intrinsic Disorder in HIV-1 Proteome

The residue wise disorder score for each protein was predicted by IUPred algorithm (Dosztanyi et al., 2005a) using the option to predict long disordered regions. We choose IUPred algorithm for disorder prediction as IUPred didn’t train on any specific dataset and hence it gives an unbiased estimate of disorder score (Dosztanyi et al., 2007). Moreover, this algorithm has been validated to reflect truly the structural status of proteins in viral genomes (Hagai et al., 2014). Based on the pairwise interaction energy score, IUPred assigns disorder scores for each amino acid (Dosztanyi et al., 2005b). Residues having a disorder score above threshold value 0.5 were considered as disordered. From these predicted disorder scores, we calculated several measures of protein disorder content. We have measured the percentage of disordered residues by calculating the total disordered residues to the total amino acid residues of the proteins. Next, the percentages of disordered residues were averaged for all proteins in the proteome.

### Prediction of Short Linear Motif (SLiMs)

The short linear motif patterns were downloaded from ELMdb^1^ (Dinkel et al., 2012). The prediction of SLiMs on HIV-1 proteome was done using in-house perl scripts. In order to predict true SLiM in OVR as well as in non-overlapping region (NOVR) we took an approach similar to Hagai et al. (2014) where, we have shuffled the entire protein sequence of each gene 1000 times and predicted SLiMs in the shuffled set of sequences. The SLiMs that were retained in both the shuffled set of sequences as well as the original type were referred to be false positives, while the SLiMs that occurred in original sequences but disappeared upon shuffling were referred to as the original or true SLiMs. We scored each SLiM according to their occurrences in the randomly shuffled sequence set, for instance if a SLiM occur 10 times out of 1000, then the probability of occurrence of that SLiM in shuffled set is 0.01. Next, we classified those SLiMs as true SLiMs which has a probability of occurrence in the shuffled set is less than 0.1. The sequences were shuffled using the Ushuffle program (Jiang et al., 2008) with seed 10^-4^. Experimentally validated SLiMs that are involved in interaction with host proteins were retrieved from the published dataset of Davey et al. (2011) and Halehalli and Nagarajaram (2015).

We calculated SLiMs which are present within intrinsically disordered residues, since they may act as potential molecular recognition features (Mohan et al., 2006). We identified true SLiMs whose positions coincided with intrinsically disordered residues and termed them as functionally potential SLiMs.

### Estimation of SLiM Conservation Level

Conservation levels of the respective true SLiM positions were estimated where a conservation score was assigned to each of the SLiMs identified. The conservation score was calculated as the ratio of the number of HIV-1 genome in which a given SLiM was retained in the same position in all orthologous genes of the respective genomes and the total number of HIV-1 genomes studied.

### Enrichment of Protein Phosphorylation Sites in HIV-1 Proteome

Prediction of protein phosphorylation (PP) sites were done using NetPhos 2.0 (Blom et al., 1999) which gives accurate predictions using neural network for serine, threonine and tyrosine residues in a given protein. It assigns a score to each serine, threonine or tyrosine residues indicating the probability of these residues of getting phosphorylated. Residues with scores greater than 0.50, indicate potential PP sites. List of experimentally validated phosphorylation sites were obtained from (Schwartz and Church, 2010).

### Statistical Analyses

Mann–Whitney *U* test was used for pairwise comparison of different factors used in this study. Spearman’s bivariate correlations were used to find out the association between factors. All of these statistical analyses were done using SPSS 13.0 package.

## Results

### OVRs of HIV-1 Genome Tend to Encode Intrinsically Disordered Residues

**FIGURE 1:**
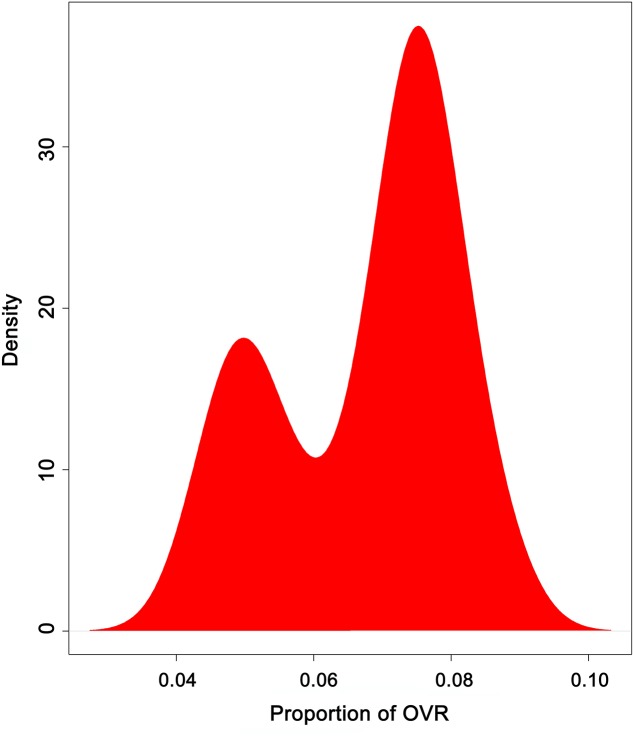
**Density distribution of proportion of OVR (*P_ov_*) across 35 HIV-1 subtypes showing the minimum and maximum values of *P_ov_***.

Previously, it was reported that the HIV-1 genome contains overlapping genesthat contribute to synonymous site conservation in the virus (Mayrose et al., 2013). Here, we identified 235 OVRs of varying lengths across 35 HIV-1 group M subtype genomes (**Supplementary Table 1**). The proportion of OVRs of a given genome was then calculated by summing up all the lengths of OVRs present in that genome, and then dividing it by the total length of the coding sequence of that genome. We observed that proportion of overlap varies from 4.8 to 8.0% of the total coding regions (**Figure 1**; **Supplementary Table 1**). The density plot (**Figure 1**) demonstrates that majority of the genomes have proportion of OVR of 8% of the total coding regions.

**FIGURE 2:**
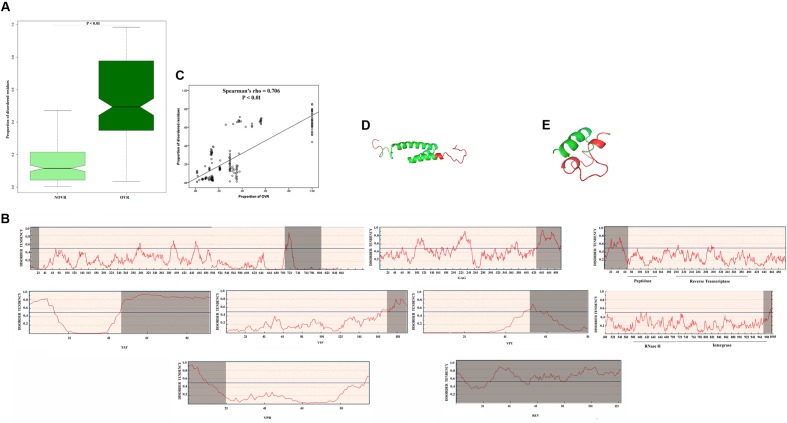
**(A)** Boxplot showing difference in distribution of proportion of intrinsically disordered residues in overlapping region (OVR) and non-overlapping region (NOVR) of 35 HIV-1 subtype genomes **(B)** Graphical representation of disorder tendency across the entire protein length of 8 proteins of HIV-1group M genome. The analysis was carried out with *gag* (BAF31333.1), *env* (BAF31340.1), *tat* (BAF31337.1), *rev* (BAF31338.1), *vif* (BAF31335.1), *vpu* (BAF31339.1), *vpr* (BAF31336.1), and *pol* (BAF31334.1) genes of a Rwandan subtype, AB253421. The four catalytic domains of *pol* gene have been pointed. The OVRs are highlighted in gray. **(C)** Scattered plot showing the correlation between proportion of OVR and proportion of disordered residues across the different genes of HIV-1 subtype genomes **(D)** Protein Databank (PDB) structure of *vpr* protein (PDB ID – 1M8L) with OVR highlighted in red **(E)** PDB structure of *vpu* protein (PDB ID – 1VPU) with OVR highlighted in red.

It was reported that viruses of eukaryotes, often encode for proteins with unstructured regions those are less constrained and more likely to accumulate mutations (Pushker et al., 2013). This may in turn expedite the advent of novel functions during the evolution of the virus (Gitlin et al., 2014). Previously, it was shown that OVR in unspliced RNA viruses encode an elevated degree of protein intrinsic disorder (PID; Rancurel et al., 2009). So, we intended to investigate whether the OVRs of HIV-1 genome have the potency to encode PID. In calculating the proportion of disordered residues within the OVRs and NOVRs of all the genes of 35 HIV-1 group M subtype genomes, it was noticed that disordered residues are significantly (*P* = 0.003) overrepresented in OVR than NOVR (**Figure 2A**). To study the effect of gene overlapping on PID more explicitly, we decided to split the data into eight HIV-1 genes (*tat, rev, gag, pol, vif, vpr, env*, and *vpu*). It was observed that except for *vpu* (which has a very ordered and stable secondary structure and a very little number of disordered residues in OVR), all other proteins encode considerable amount of PID in OVR as compared to that in NOVR (**Figure 2B**). In addition, we calculated the proportion of intrinsically disordered residues for each gene of 35 HIV-1 subtype genomes. It was observed that the proportion of OVRs of each gene shared a strong, significant positive correlation with the proportion of predicted intrinsically disordered residues in the protein encoded by the corresponding genes (**Figure 2C**).

Moreover, OVR stretches are short and terminal in nature, and short sequences are predicted to be highly disordered. We next decided to investigate whether the increase of structural disorder in OVR is independent of the length of the protein. We did a linear regression analysis taking total disordered residues in a given protein and its length as independent covariates and OVR length as dependent variable. The result of the multiple linear regression analysis is represented in **Table 1**. It could be interpreted from the given multivariate linear regression that increase of intrinsic disorder in OVR is independent of protein length.

**Table 1:** Multivariate linear regression analysis between length of overlapping regions (OVRs) (independent variable) and disordered residues in OVR, total protein length.

Covariates	β	*P*-value
Number of disordered residues	3.82	1.6 × 10^-4^
Total protein length	4.99	10^-6^


Next, we planned to validate whether regions predicted by IUPRED is actually disordered or not. For this purpose we retrieved the structure of individual HIV-1 proteins from Protein Databank (PDB) and tried to ascertain whether the OVR in individual proteins are actually disordered. It was previously shown by Le Gall et al. (2007) that the missing residues (unobserved residues) in X ray crystallographic structures could be structurally disordered, due to their highly dynamic atom positions which vary significantly over time. Taking this into consideration, we have mapped the OVR in PDB structure of HIV-1 proteins, the result of which are delineated in **Supplementary Table 2**. It was observed that OVRs are overrepresented in the “unobserved” or missing residue category of PDB structure. Thus, it is reasonable to assume that OVR residues could be highly dynamic and its structure could not be deciphered using standard X-Ray crystallographic protocols. Moreover, the structure of *vpr* and *vpu* are delineated in **Figures 2D,E** with their OVR highlighted in red. It could be observed that the highlighted region, i.e., OVR are structurally more flexible.

### OVRs Facilitate Enrichment of SLiMs and PPs in HIV-1 Genome

**FIGURE 3:**
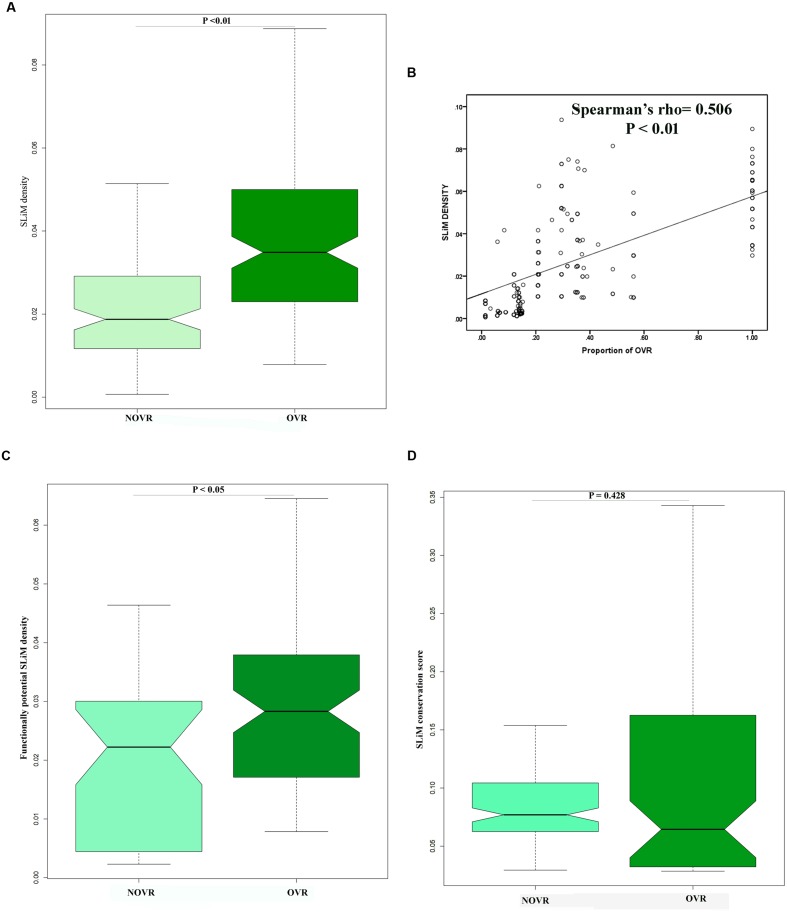
**(A)** Boxplot showing the distribution difference of true short linear motifs (SLiM) density between OVR and NOVR along with significance level indicated as *P*-value of Mann–Whitney *U* test. **(B)** Scattered plot showing the correlation between proportion of OVR and true SLiM density across the different genes of HIV-1 subtype genomes **(C)** Boxplot showing the distribution difference of functionally potential SLiM density between OVR and NOVR along with significance level indicated as *P*-value of Mann–Whitney *U* test. **(D)** Boxplot showing no significant distribution difference of conservation score of SLiM between OVR and NOVR along with significance level indicated as *P*-value of Mann–Whitney *U* test.

In the previous section, we observed that OVR encodes significant amount of PID. It has been previously reported that PID plays pivotal role in protein–protein interactions (Uversky, 2013). Therefore, we were curious to investigate whether OVRs play any role in virus–host interaction or not. In the recent years, there are considerable volume of researches that discovered a profound role of SLiMs in viral proteome that readily facilitate virus host protein–protein interaction (Hagai et al., 2014). Thus, preponderance of these types of motifs in a given viral sequence often acts as an indicator of its involvement in protein–protein interaction. Henceforth, we tried to ascertain whether OVRs in HIV-1 genome contains SLiMs to initiate virus–host interaction. In this context, we intended to find out whether OVRs, apart from encoding structural disorder are also involved in encoding SLiMs that could be essential for host–viral interactions. We scanned all the genes involved in overlapping across 35 HIV-1 group M genomes and figured out the position of different SLiMs along the entire protein length. Similarly, we shuffled individual protein sequences and figured out the probability of occurrence of SLiMs in the shuffled set. The SLiMs that occurred at a low frequency or does not occur at all in shuffled set were considered as true SLiMs. It was observed that OVRs are enriched in true SLiMs as compared to NOVR which has a depleted density of true SLiMs. (**Figure 3A**). Moreover, it was observed that the proportion of OVRs of a gene shares a significantly positive correlation with the SLiM density of the concerned genes (**Figure 3B**). We next asked whether SLiMs residing in OVRs were functionally more important than the ones residing in NOVRs. Previously, Hagai et al. (2014) determined the functional potency of SLiMs by calculating the number of SLiMs residing in disordered region of proteins. They have proposed that SLiMs, residing within locally flexible disordered regions are more suitable to participate in protein–protein interactions and thus these SLiMs are termed as functionally potential SLiMs. Similar to their analysis, we also computed densities of functionally potential SLiMs (SLiMs localizing within structurally disordered residues of proteins) in OVR as well as in NOVRs of HIV-1 genomes. It was observed that densities of functionally potential SLiMs were far higher in the OVR as compared to the NOVR (**Figure 3C**).

We further compiled a dataset of experimentally validated interaction SLiMs that are reported to interact with host proteome. We retrieved the list of genes and their corresponding interaction SLiMs **Supplementary Table 3a**), and found out whether these SLiMs are located within OVR or NOVR of that gene. Out of 71 SLiM-gene pair only 21 unique SLiM-gene pairs were observed to be matching with our true SLiM dataset. It was observed that interacting SLiMs were enriched in OVR for *tat* and *rev* proteins, *gag* protein also contained a few experimentally validated SLiMs in their OVR. In contrast, *vif, vpu* and *vpr*, and *env* have all their experimentally validated SLiMs in NOVR (**Supplementary Table 3b**).

Next we intended to find out whether the SLiMs discovered in the OVR of HIV-1 proteins are well conserved in their position across all subtypes of HIV-1 genome. For this, we have estimated the conservation level of each SLiMs located within OVRs as well as in the NOVRs. It was observed that there was no significant difference of conservation level between SLiMs located in the OVR to that in the NOVR **Figure 3D**. This may be due to higher intrinsic disorder content of OVR which relaxes the level of purifying selection on these SLiMs.

**FIGURE 4:**
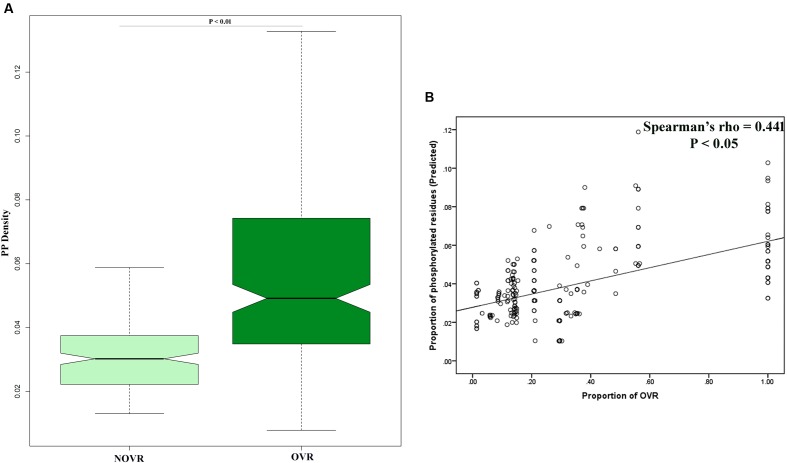
**(A)** Boxplot showing the distribution difference of protein phosphorylation (PP) density between OVR and NOVR along with significance level indicated as *P*-value of Mann–Whitney *U* test. **(B)** Scattered plot showing the correlation between proportion of OVR and PP density across the different genes of HIV-1 subtype genomes.

Another potential indicator of virus–host interaction is PTMs; Hundt et al., 2013). Precisely, among the different PTMs that take place *in vivo*, PP is one of the most important and well studied types of PTM. In previous works, PP has been shown to play a pivotal role in HIV-1 interactome (Francis et al., 2011). Therefore, we were interested in exploring the role of gene overlapping in effecting PP across the protein length. In order to ascertain this we have mapped the OVR of each gene and calculated the PP density of OVRs as well NOVRs. It was observed that the PP densities of OVRs were far greater than that of NOVRs (**Figure 4A**). It was also observed that the proportion of gene overlapping correlated with a fraction of PP density of a given gene (**Figure 4B**). We have confirmed our results using experimentally validated PP sites, the results of which are delineated in **Supplementary Table 4**.

### Role of PID and OVR in Acquisition of SLiMs and PPs HIV-1 Genome

**FIGURE 5:**
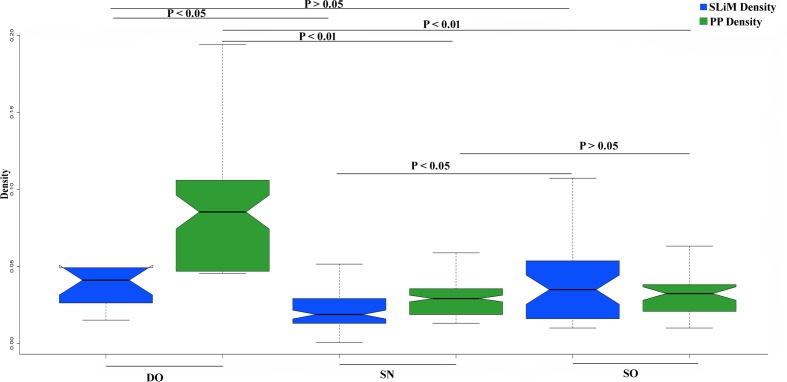
**The distribution difference of PP and SLiM Density between DO, SN, and SO group with *P-values* indicating the level of significance of Mann-Whitney *U* test**.

We have found that OVRs attributed to the overrepresentation of SLiMs and PPs in HIV-1 genome. Now, it was also reported that SLiMs and PP sites encompass affluence of disordered residues (Iakoucheva et al., 2004; Meszaros et al., 2012). Thus, we have asked whether the enrichment of SLiMs and PP sites are by-products of an abundance of intrinsic disorder in the respective OVRs or OVRs by virtue harbors more SLiMs and PP sites as compared to NOVRs. In other words, we were interested to find out whether the overrepresentation of SLiMs and PPs in OVRs are independent of PID. For this, divided each HIV-1 subtype proteome into three categories- (i) OVRs with 80% or more of its residues in disordered state (DO) (ii) OVRs with 80% of its residue in the structured state (SO) (iii) NOVR with 80% or more of its residue in structured state PID (SN). Our hypothesis, in this context was that DO would entail elevated degree of SLiMs and PPs as compared to SO and SN, since it is highly disordered as well as overlapping in nature. In addition, SO would entail an elevated density of PP and SLiM as compared to SN if the occurrences of PPs and SLiMs are mutually exclusive of PID. This is because the phenomenon of gene overlapping will itself be responsible for enrichment SLiMs and PPs in SO, whereas SN (where there is no genetic overlap) would definitely confer depleted density of SLiMs and PPs. On the other hand, the absence of significant difference of PP and SLiM density between SO and SN would reflect that PID is the reason behind the elevated densities of PPs and SLiMs in the OVR and OVR doesn’t have any significant impact over PP and SLiM density. Finally, we estimated the SLiM and PP density for these three categories separately, the result of which are delineated in (**Figure 5**). It could be observed that SLiM content is higher in SO as compared to SN which reflects that acquisition of elevated frequency of SLiMs in the OVRs is mutually exclusive of PID. On the contrary occurrence of PP was not mutually exclusive of PID since there was no significant difference of PP density between SO and SN groups. Hence, it could be interpreted that although acquisition of SLiMs in OVR is exclusive of PID, but enrichment of PP in OVR was mainly due to increased disorder content of OVRs.

## Discussion

Gene overlapping is a common phenomenon in viral genomes and has several important implications and significances in viral evolution (Hughes et al., 2001; Guyader and Ducray, 2002; Pavesi, 2006; Zhao et al., 2007). Other than genome size evolution, gene overlapping might increase selective constraints at synonymous sites of viral genes (Ngandu et al., 2008). Our study is focused to investigate the role of OVR in viral pathogenicity. In order to do so, we have analyzed the properties of OVR encoded proteins and noticed that they encode a considerable amount of structural disorder as compared to NOVRs. This observation is in agreement with a previous report by Rancurel et al. (2009), where it has been observed that OVRs of unspliced RNA viruses encodes an elevated degree of PID. Their work put forward a noble explanation behind such an observation. Since disordered proteins are under weaker selection pressure than the ordered ones (Brown et al., 2002), they proposed that PID encoded by OVRs might balance out the excessive amount of evolutionary constraints imposed over them due to their dual coding natures. As a result, the protein portions encoded by OVRs are structurally flexible in order to shed off their structural complexities without losing their key functions. Earlier, studies on amino acid compositions of OVRs of viral proteins revealed a strong bias toward highly degenerate codons which are found to encode PID (Rancurel et al., 2009). However, OVRs were previously shown to be evolutionarily conserved as compared to the NOVRs (Simon-Loriere et al., 2013) and on the contrary disordered residues have been previously shown to evolve faster and are under weaker selection pressure (Gitlin et al., 2014). Thus, apparently it is surprising that these evolutionarily conserved regions encode sufficient amount of PID. PID often mediates massive protein–protein interaction that could add functional constrain to the evolution of regions encoding PID (Brown et al., 2002; Chen et al., 2006; Bellay et al., 2011). Moreover, enrichment of phosphorylation sites in intrinsically disordered regions could also make these regions evolutionarily conserved as site of PP may involve in many different molecular functions (Maathuis, 2008). Thus, intrinsically disordered regions sometimes show higher level of conservation as compared to structured regions and so OVRs in spite of encoding elevated degree of protein intrinsic disorder could be evolutionarily constrained. In order to investigate whether PID encoded by OVR are indeed responsible for massive protein–protein interaction, we decided to explore whether there is (i) over-representation of SLiMs (ii) abundance of PP sites in OVRs. In agreement to our hypothesis we indeed noticed a significant over-representation of PP and SLiMs in the OVRs.

It is already known that viruses with extremely diminished genome size encode proteins of short length. Thus, structured domains are seldom in viral proteome (Rappoport and Linial, 2012). Rather, extensive usage of SLiMs and in viruses facilitate hijacking of host cellular machineries and evade host immune system (Hagai et al., 2014). Again, interaction motifs are observed to be rapidly evolving and thus facilitate quick rewiring of virus–host interaction network (Jackson et al., 2003). Given the fact that RNA viruses accumulate more mutations than DNA viruses, tolerability of SLiMs to mutations thus facilitate a lesser extent of disruption of important interactions with the host (Hagai et al., 2014). In other words, SLiMs have shorter interfaces as compared to globular domains and are involved in transient or conditional interactions with the host (Davey et al., 2012). Henceforth they are more suitable to mimic multiple host peptides by virtue of their versatile interaction modes (Duro et al., 2015). This promiscuously is highly favored by occurrences of conformational flexibility of regions surrounded by these peptide motifs (Fuxreiter et al., 2007). As a result SLiMs in viruses are often localized into short stretches of disordered residues. PID thus imparts additional functional potency to these peptide motifs. Hence, we have determined the distribution of functionally potent SLiMs in OVRs and NOVRs of HIV-1 genome. Consequently, by analyzing distribution of predicted SLiMs, it was observed that SLiMs in the OVR tend to be more functionally potential (Beauclair et al., 2013). In addition to SLiMs, OVRs was also observed to contain significant amount of predicted PP sites compared to NOVRs. Over-representation of PP sites in OVRs further reflects the importance of gene overlapping in HIV-1 genomes. Previously, there has been range of studies focusing on the role of PP in the development of productive infection cycle by viruses within their host (Keck et al., 2015). PPs of HIV-1 proteins have been found to be associated with nuclear transport, replication, repair, and transcription (Idriss et al., 1999; Francis et al., 2011; Kudoh et al., 2014). For instance, HIV-1 integrase has been shown to be directly phosphorylated by host kinases in order to integrate into the host genome efficiently (Manganaro et al., 2010). On the other hand extensive phosphorylation of viral proteins might in favor of the pathogen and increase the affinity of a given protein to participate in protein–protein interactions with the host (Keck et al., 2015). Thereby, accumulation of PP residues within OVRs also suggests its importance in development of viral infection and increasing viral pathogenicity.

Our study also sought to address whether OVRs by virtue accumulate SLiMs and PPs or it is the conformational flexibility of intrinsically disordered regions within OVRs that drives the increased frequency of SLiMs and PP residues in these regions. Our observation undoubtedly reveals that the impacts of PID and OVR in increasing SLiM density over these regions are mutually exclusive. However, the enrichment of PP sites in OVR is a byproduct of elevated structural disorder in these regions. This finding adds another interesting dimension to our study.

Hence, our work sheds light on the significance of overlapping genes in the virulence of HIV-1. Thus, it paves inroads for future studies to explore the therapeutic tools against these culprit regions to overcome AIDs.

## Author Contributions

All authors contributed to the design of experiments, the interpretation of data, and drafting of the manuscript. Experiments were performed by DS.

## Conflict of Interest Statement

The authors declare that the research was conducted in the absence of any commercial or financial relationships that could be construed as a potential conflict of interest.

## Footnotes

1 http://elm.eu.org
